# Alarming antibiotics resistance of *Helicobacter pylori* from children in Southeast China over 6 years

**DOI:** 10.1038/s41598-022-21661-y

**Published:** 2022-10-22

**Authors:** Xiaoli Shu, Diya Ye, Chenmin Hu, Kerong Peng, Hong Zhao, Huamei Li, Mizu Jiang

**Affiliations:** 1 Gastrointestinal Laboratory, The Children’s Hospital, Zhejiang University School of Medicine, National Clinical Research Center for Child Health, National Children’s Regional Medical Center, Hangzhou, China; 2 Department of Gastroenterology, The Children’s Hospital, Zhejiang University School of Medicine, National Clinical Research Center for Child Health, National Children’s Regional Medical Center, Hangzhou, China; 3 Pediatric Endoscopy Center, The Children’s Hospital, Zhejiang University School of Medicine, National Clinical Research Center for Child Health, National Children’s Regional Medical Center, Hangzhou, China

**Keywords:** Antimicrobial resistance, Clinical microbiology

## Abstract

The increasing rates of antibiotic resistance in *Helicobacter pylori* (*H. pylori*) are a major concern of the decreasing eradication rate. Large-scale and long-period studies on antimicrobial susceptibility of *H. pylori* in children are limited. This study aimed to describe the temporal changes of antibiotic resistance among children in southeast China. Gastric biopsies obtained from children were cultured for *H. pylori* from 2015 to 2020. Susceptibility to clarithromycin (CLA), amoxicillin (AML), metronidazole (MTZ), furazolidone (FZD), tetracycline (TET) and levofloxacin (LEV) was tested. Data from 2012 to 2014 reported previously were obtained for comparing the change in temporal trends of antibiotic resistance. A total of 1638 (52.7%) *H. pylori* strains were isolated from 3111 children recruited. The resistance rates to CLA, MTZ and LEV were 32.8%, 81.7% and 22.8%, respectively. There were 52.9% strains resistant to single resistance, 28.7% to double resistance, and 9.0% to triple resistance. The total resistance rate and resistance rates to CLA, MTZ, LEV, CLA + LEV and CLA + MTZ + LEV increased annually in a linear manner. All resistant patterns except single resistance increased obviously from 2015 to 2017 and 2018 to 2020 compared to that from 2012 to 2014. Double resistance to CLA + MTZ increased significantly with age. The resistance rate to CLA and triple resistance to CLA, MTZ and LEV increased in children with prior *H. pylori* treatment than that from children without prior treatment. The antibiotic resistance rates of *H. pylori* were high in a large pediatric population in southeast China from 2015 to 2020. Individual treatment based on susceptibility test is imperative and optimal regimens should be chosen in *H. pylori* eradication therapy.

## Introduction

*Helicobacter pylori* (*H. pylori*) infection is a worldwide, usually lifelong disease that is found both in children and adults. The bacterium is the major cause of chronic active gastritis, peptic ulcers, and is the strongest known risk factors for gastric cancer and mucosa-associated lymphoid tissue lymphoma^1^. Though only 1–3% of infected individuals will develop malignant complications, the mortality rate of gastric cancer in China ranks third among malignant tumors for males and second for females, making it a serious public health issue that needs to be solved urgently^2^. And there is an abundance of evidence to suggest that *H. pylori* eradication reduces the risk of gastric cancer as well as its precancerous lesions^3^. Accordingly, all major gastroenterological societies recommend that *H. pylori* be eradicated in individuals who test positive, especially in adults^4–6^.

*H. pylori* infection in children and adolescents rarely develop complications of infection such as peptic ulcer, atrophic gastritis and gastric cancer compared with adults. In addition, children with *H. pylori* infection have a certain spontaneous clearance rate, and the reinfection rate may be higher than that in adults after eradication. Therefore, it is not recommended to detect *H. pylori* routinely in children under 14 years of age and *H. pylori* “test and treat” strategy is only suggested in children with peptic ulcer disease and dyspepsia after endoscopic evaluation because of great benefits achieved in children with peptic ulcer after *H. pylori* eradication^6–8^.

Nevertheless, treatment options in the pediatric population are more limited as the fact that some antibiotics are not licensed for the pediatric population, such as tetracycline (TET), furazolidone (FZD), and levofloxacin (LEV), which are appropriate for use in adults with *H. pylori* infection^6^. The classical recommended first-line eradication regimens of *H. pylori* in children are mainly triple therapy consisting of one proton-pump inhibitor plus two antibiotics chosen from amoxicillin (AML), clarithromycin (CLA), and metronidazole (MTZ) for a duration of 14 days^7,9^.

Given the limited antibiotics appropriate for *H. pylori* eradication in children and increased antibiotic resistance worldwide, the updated international consensus or guidelines issued by authoritative groups strongly recommend choosing antibiotics based on local resistance patterns to increase the efficacy of eradication^7–9^. However, limited access of endoscopic examination and the fastidious culture of *H. pylori*, the antimicrobial susceptibility tests are not available universally. The data for resistance rates obtained from regional or population-specific reports is of great importance in guiding the effective treatment regimens. This study aimed to determine the resistance rates and patterns of *H. pylori* in children with upper gastrointestinal symptoms from 2015 to 2020 and to monitor the temporal trends of antibiotic susceptibility over recent years in a National Clinical Research Center for Child Health in Southeast China.

## Materials and methods

### Patients

This study was performed at the Children’s hospital, Zhejiang University School of Medicine, the National Clinical Research Center for Child Health, and the largest tertiary hospital for pediatric health care in Zhejiang province from March 2015 to December 2020. The subjects included children who presented with upper gastrointestinal symptoms and had undergone upper gastrointestinal endoscopy. Then culture for *H. pylori* was performed and antibiotic susceptibility of *H. pylori* to CLA, AML, MTZ, LEV, FZD and TET were tested if the culture was positive. If endoscopy was performed more than one time in a given child, only the first one was included. Demographic data (including age and sex) and history of *H. pylori* eradication were obtained from each enrolled child. Data from 2012 to 2014 reported previously were obtained for comparing the change in temporal trends of antibiotic resistance^10^.

### Ethics approval

This study was approved and need of informed consent was exempted by the Ethics Committee of the Children’s hospital, Zhejiang University School of Medicine (No.2021-IRB-078). All methods were performed according to the relevant guidelines.

### Isolation and culture of *H. pylori* strains

Gastric mucosa biopsies were preserved in sterile vials containing 0.5 ml brain–heart infusion broth (Oxoid, Basingstoke, UK) supplemented with 20% glycerol and stored at 4 °C until transfer with dry ice later the same day to Zhiyuan Medical Inspection Institute, Hangzhou, that is a professional platform founded in 2005 in Hangzhou for *H. pylor*i isolation, culture and antibiotic susceptibility test. If biopsies in preservation solution are not transferred timely, they are kept at − 80 °C till the transfer within a week. The homogenate of stomach biopsy specimens was inoculated onto Columbia agar (Oxoid, Basingstoke, UK) plates supplemented with 5% fresh defibrinated sheep blood and antibiotics mixture including amphotericin B, vancomycin and polymyxin, then kept at 37 °C under microaerophilic conditions (5% O_2_, 10% CO_2_ and 85% N_2_) for 3–4 days. Colonies displaying typical *H. pylori* morphology were selected and identified by urease, oxidase, and catalase activity testing.

### Antibiotic susceptibility tests

Susceptibility of *H. pylori* to six antibiotics (CLA, AML, MTZ, LEV, FZD and TET) was tested via agar dilution method using reference standards obtained from the National Institutes for Food and Drug Control. Two microliter suspensions (6 × 10^8^ CFU/ml) of each isolate from grown well colonies in 0.9% saline solution were inoculated onto Columbia agar that included 5% sheep blood and a single antibiotic and incubated at 37 °C for 2–3 days under microaerophilic conditions. Based on the Clinical and Laboratory Standards Institute (CLSI) document M100-S18 and other previously published data, the resistance break points to CLA, AML, MTZ, LEV, FZD and TET were set at ≥ 1, ≥ 2, ≥ 8, ≥ 2, ≥ 2, and ≥ 2 μg/ml, respectively^11–14^. The *H. pylori* strain ATCC 43504 was used as the quality control of the susceptibility tests. All the tests were conducted at Zhiyuan Medical Inspection Institute, Hangzhou.

### Statistical analysis

Statistical analyses were carried out using SPSS statistical software package version 19.0 (SPSS Inc., Chicago, IL, USA). The outcome variables were described as frequency counts and were presented as rates (%) of single and combined resistance to three antibiotics. Differences in resistance rates between different gender, age, years, time periods and antibiotic resistance groups were analyzed with a chi-squared $\left( {\chi^{2} } \right)$ test. Linear regression model was used to assess changes in rate over time. A *P* value < 0.05 was considered statistically significant.

## Results

### Epidemiological data of patients

Gastric biopsy and bacteriological culture were performed in 3111 children who underwent endoscopy from March 2015 to December 2020. There were 1835 boys and 1276 girls, yielding a male-to-female ratio of 1.4:1. The mean age (± SD) of all children was 9.2 ± 3.1 years. Boys were 9.2 ± 3.1 years and girls were 9.2 ± 3.0 years.

### *H. pylori* culture

A total of 1638/3111 (52.7%) *H. pylori* strains were isolated from 2015 to 2020. There were 1003 boys and 635 girls with a male-to-female ratio of 1.6:1, which was higher than that in culture-negative children (1.3:1, *P* < 0.01) (Table 1). The mean age (± SD) of culture-positive children was 9.4 ± 2.9 years, which was older than culture-negative children (9.1 ± 3.2, *P* < 0.01) (Table 1). The proportion of *H. pylori* culture-positive was slightly different in the three age groups, in descending order: the 7–12 years group, the 13–18 years group and the 1–6 years group (*P* < 0.05) (Table 1).

**Table 1:** Demographic characteristics and *H. pylori* culture of the 3111 subjects from 2015 to 2020.

Variables	*H. pylori* positive	*H. pylori* negative	Total	*P* value
**Gender (*n* [%])**				
Male	1003 (54.7)	832 (45.3)	1835	
Female	635 (49.8)	641 (50.2)	1276	0.007
**Age (year, mean ± SD)**	9.4 ± 2.9	9.1 ± 3.2	9.2 ± 3.1	0.006
**Age groups, year (*n* [%])**				
1–6	400 (49.0)	417 (51.0)	817	
7–12	1007 (54.4)	845 (45.6)	1852	
13–18	231 (52.3)	211 (47.7)	442	0.035
Total	1638 (52.7)	1473 (47.3)	3111	−

### *H. pylori* antibiotic susceptibility

After *H. pylori* strains were isolated, antibiotic susceptibility testing was performed subsequently. Of the 1638 *H. pylori* strains, only 9.4% (154/1638) were sensitive to all antibiotics. The total resistance rates of *H. pylori* to CLA, MTZ and LEV were 32.8% (538/1638), 81.7% (1339/1638), and 22.8% (373/1638), respectively (Table 2). And there was no resistant strain to AML, FZD and TET. 52.9% (866/1638) strains were single resistance to CLA, MTZ or LEV, and 37.7% (618/1638) strains were resistant to more than one antibiotic, 28.7% (470/1638) for double resistance, and 9.0% (148/1638) for triple resistance. The predominant patterns of double resistance were CLA + MTZ (16.4%) and MTZ + LEV (10.6%), followed by CLA + LEV (1.8%).

**Table 2:** Results of antibiotic susceptibility tests of 1638 *H. pylori* strains isolated from 2015 to 2020.

Susceptibility test results	Number of strains	Resistance rate (%)
**Total resistance**		
CLA	538	32.8
MTZ	1339	81.7
LEV	373	22.8
**Single resistance**		
CLA	93	5.7
MTZ	750	45.8
LEV	23	1.4
**Double resistance**		
CLA + MTZ	268	16.4
CLA + LEV	29	1.8
MTZ + LEV	173	10.6
**Triple resistance**		
CLA + MTZ + LEV	148	9.0

CLA, clarithromycin; MTZ, metronidazole; LEV, levofloxacin.

### Temporal trends in antibiotic susceptibility

**Figure 1:**
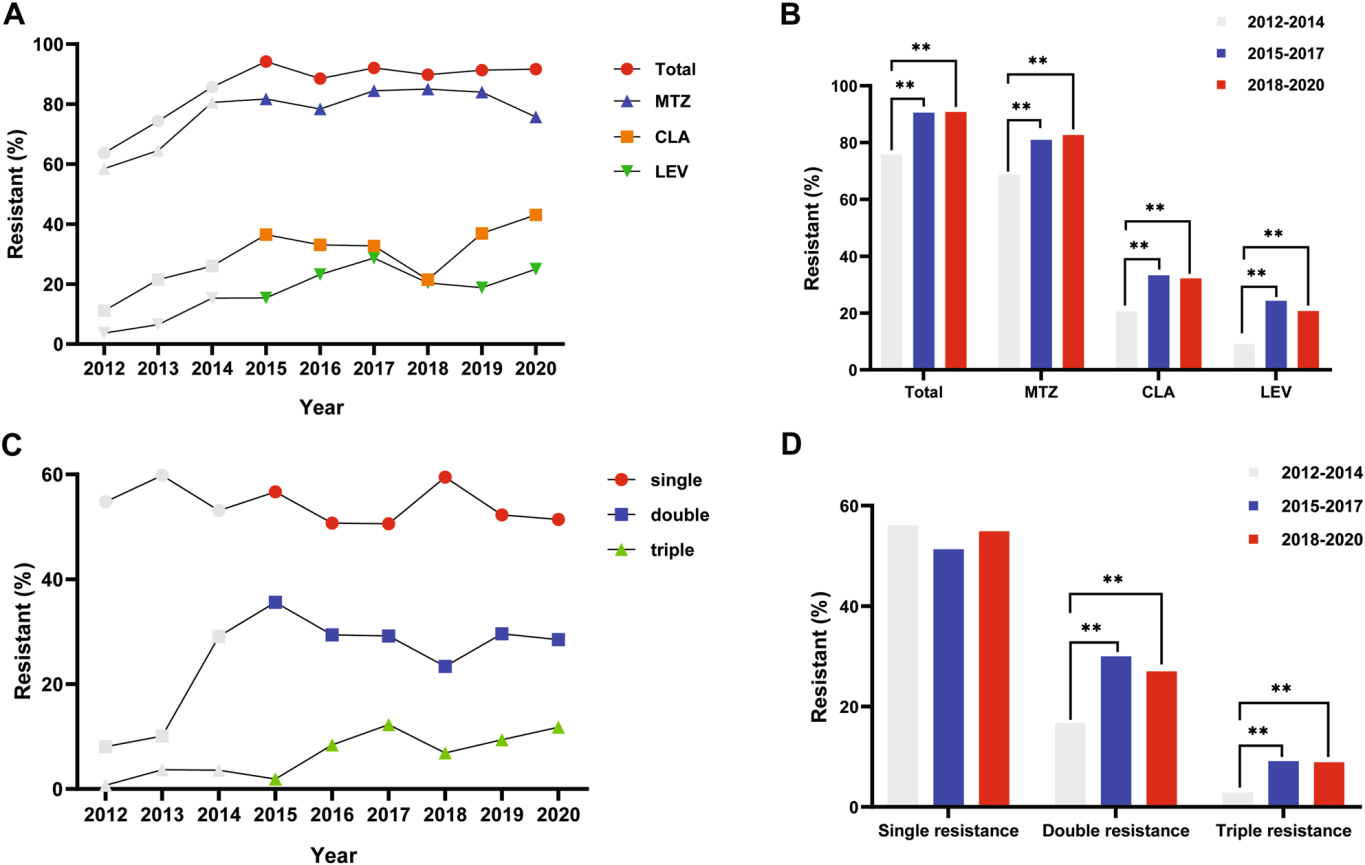
Antibiotic resistance of *H. pylori* to clarithromycin, metronidazole and levofloxacin according to year. (**A**) The total resistance and resistance to clarithromycin, metronidazole and levofloxacin annually from 2012 to 2020. (**B**) The total resistance and resistance to clarithromycin, metronidazole and levofloxacin in different time periods, 2012–2014, 2015–2017 and 2018–2020. (**C**) Single, double and triple resistance annually from 2012 to 2020. (**D**) Single, double and triple resistance in different time periods, 2012–2014, 2015–2017 and 2018–2020. CLA, clarithromycin; MTZ, metronidazole; LEV, levofloxacin. **P < 0.01.

**Figure 2:**
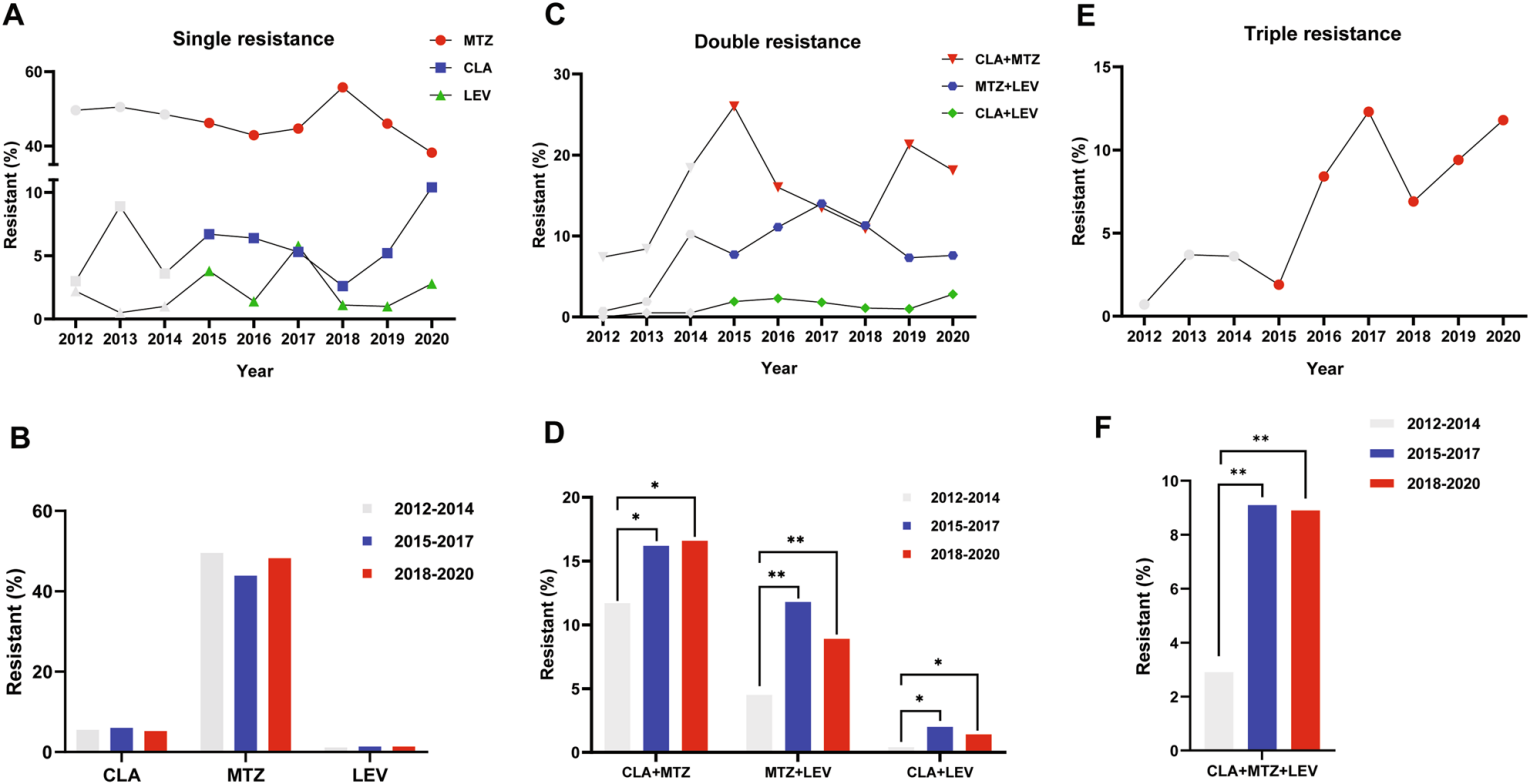
Antibiotic resistance patterns of *H. pylori* to clarithromycin, metronidazole and levofloxacin according to year. (**A**) Single resistance to clarithromycin, metronidazole and levofloxacin annually from 2012 to 2020. (**B**) Single resistance to clarithromycin, metronidazole and levofloxacin in different time periods, 2012–2014, 2015–2017 and 2018–2020. (**C**) Double resistance annually from 2012 to 2020. (**D**) Double resistance in different time periods, 2012–2014, 2015–2017 and 2018–2020. (**E**) Triple resistance annually from 2012 to 2020. (**F**) Triple resistance in different time periods, 2012–2014, 2015–2017 and 2018–2020. CLA, clarithromycin; MTZ, metronidazole; LEV, levofloxacin. *P < 0.05, **P < 0.01.

We found that the *H. pylori*-resistance rate was extremely high over 6 years, from 2015 to 2020.To investigate the change in temporal trends of antibiotic resistance, our previously reported data from 2012 to 2014 were involved for further analysis^10^. We confirmed the *H. pylori*-resistance rate rose gradually from 2013 or 2014 and maintained at a high level (Figs. 1, 2). With a linear regression model, the total resistance increased by 2.8% each year relative to that in the preceding year (*P* < 0.01) while the resistance to CLA, MTZ and LEV increased by 2.7%, 2.3% and 2.4% (*P* < 0.05, *P* < 0.05 and *P* < 0.01 respectively). Double resistance to CLA + LEV increased by 0.2% each year (*P* < 0.05) and triple resistance to CLA, MTZ and LEV increased by 1.3% each year (*P* < 0.01). Double resistance to CLA + MTZ or MTZ + LEV, and single resistance to CLA, MTZ or LEV did not follow a linear pattern. When grouped into 3-year time periods, all resistant patterns increased obviously from 2015 to 2017 and 2018 to 2020 compared with that from 2012 to 2014, except single resistance to CLA, MTZ or LEV (Figs. 1, 2).

### Factors associated with antibiotic resistance

Gender was not associated with resistance to any antibiotic. Double resistance to CLA + MTZ increased significantly in the 7–12 years (17.6%) and the 13–18 years (18.2%) groups compared with the 1–6 years group (12.3%) (*P* < 0.05) (Table 3). The strains isolated from children with prior *H. pylori* treatment were more likely to be resistant to CLA than that from children without prior treatment (44.8% vs. 31.9%, *P* < 0.01). Similarly, triple resistance to CLA, MTZ and LEV increased in children with prior *H. pylori* treatment (15.2% vs. 8.8%, *P* < 0.05) (Table 3).

**Table 3:** Factors associated with antibiotic resistance of *H*. *pylori* from 2015 to 2020.

Resistance rate (%)	Gender Male (*n* = 1003) Female (*n* = 635)	Age (years) 1–6 (*n* = 400) 7–12 (*n* = 1007) 13–18 (*n* = 231)	Prior treatment No (*n* = 1513) Yes (*n* = 125)
**Total resistance**			
CLA	33.1, 32.4	30.8, 34.0, 31.6	31.9, 44.8^a^
MTZ	81.2, 82.7	79.5, 82.3, 83.1	81.3, 87.2
LEV	22.3, 23.5	25.0, 22.7, 19.0	22.7, 23.2
**Single resistance**			
CLA	5.8, 5.5	6.0, 5.7, 5.2	5.6, 6.4
MTZ	45.3, 46.6	46.5, 45.0, 48.1	45.9, 44.0
LEV	1.3, 1.6	1.8, 1.4, 0.9	1.5, 0
**Double resistance**			
CLA + MTZ	16.8, 15.7	12.3, 17.6, 18.2^b^	28.6, 29.6
CLA + LEV	1.9, 1.6	2.5, 1.6, 1.3	15.9, 21.6
MTZ + LEV	10.5, 10.7	10.8, 10.6, 10.0	1.8, 1.6
**Triple resistance**			
CLA + MTZ + LEV	8.7, 9.6	10.0, 9.1, 6.9	8.8, 15.2^b^

CLA, clarithromycin; MTZ, metronidazole; LEV, levofloxacin.

^a^*P* < 0.01.

^b^*P* < 0.05.

## Discussions

Success of *H. pylori* eradication is mainly dependent on the usage of susceptible antibiotics^15^. However, antimicrobial susceptibility testing for *H. pylori* is not universally available. Therefore, the choice of an effective empirical eradication therapy is based on region and population-specific antibiotic resistance patterns. In the present single-center study, we conducted a large patient-based investigation to evaluate the resistance rates of six most common antibiotics for *H. pylori* eradication in southeast China from 2015 to 2020. Our study indicated that the resistance rates of *H. pylori* to CLA, MTZ and LEV were extremely high from 2015 to 2020 and found a linear increase in most of resistance patterns over the past 9 years.

First concern is the rapid development of high rates of resistance to CLA, which is an importance component of first-line treatment regimens. In our study, the overall resistance to CLA increased from 11.1% in 2012^10^ to 43.1% in 2020 in our center, by 2.8% each year, and remained sustainably above 15% from 2013 to 2020, which is a threshold of CLA resistance rate for the standard triple therapy in Maastricht IV/Florence consensus report^5^. It was higher than that in most regions worldwide^16^ but similar in other areas of China^17^. It is reported that CLA resistance correlated with treatment failure^18,19^. Among the patients who received CLA-containing regimens, strains with CLA resistance were significantly higher in those with failed eradication^18^. Unfortunately, the effects of antibiotic resistance on *H. pylori* eradication efficacy could not be evaluated because the treatment regimens and outcomes were not obtained completely in this study. In the future, we’ll follow up the subsequent treatment regimens and eradication efficacy.

The prescription of CLA in children during the last decade for respiratory tract infections might contribute to a high resistance rate of *H. pylori* to CLA^20^. There is statistical significance between macrolide and quinolone consumption in the community and corresponding *H. pylori* resistance in European countries^21,22^. Data at a population level nationwide revealed a significant upward trend of antibiotic consumption including macrolides in China’s tertiary hospitals from 2011 to 2015, and then the overall consumption of macrolides decreased slightly from 2015 to 2017^23,24^. It seems to be consistent with the trend of the resistance rate of *H. pylori* to CLA from 2012 to 2018 (Fig. 1A).

MTZ is also one of the earliest and the most common antibiotics used for *H. pylori* eradication. However, the prevalence of MTZ resistance was high worldwide, especially in China because of the increased prescription of MTZ, especially for dental infection and parasitic infection. In addition, many clinicians mistakenly believe that in vitro resistance cannot impede the use of MTZ because the increasing dosage and treatment duration will help to increase the eradication rate. These might result in extremely high resistance rate of MTZ in our area (81.7%), which have remained at an elevated level since 2014 (Fig. 1). Even more, double resistance to CLA + MTZ was higher (16.4%) than 15%, and 77.3% (416/538) of the strains were resistant to MTZ in CLA resistant isolates. According to the updated consensus reports, bismuth-containing quadruple therapies with a proton-pump inhibitor, bismuth and a combination of two antibiotics, among FZD, TET, MTZ and AML, are the recommended first-line treatment in those regions with high (> 15%) dual CLA and MTZ resistance^5,6^. But TET, and FZD which are used for *H. pylori* eradication in adults are relatively contradicted in children because of potential side effects and the evidences supporting this regimen in children and adolescents are limited^25^. The use of TET can be considered instead of AMO in children older than 8 years if the strain is resistant to CLA in the case of penicillin allergy in the updated ESPGHAN/NASPGHAN guideline^7^. However, TET and FZD are not licensed to be used in children in China and Japan^8,26^.

LEV is usually contraindicated in children younger than 18 years because of potential severe side effects, though it has been extensively studied in eradicating *H. pylori* and been proven to be effective in adults. The overall resistance rate of *H. pylori* to LEV also increased over time in our studies though it was lower than that in adults^27^. As *H. pylori* infection is usually acquired in childhood mainly through intra-family transmission, the high LEV resistance in children might be explained by the transmission of LEV-resistant strains from parents to children.

There was no AML resistant strain in our study, indicating scarce incidence of this antibiotic resistance of *H. pylori*, which was in concordance with previous studies^10,17,28^. Some AML-resistant strains of *H. pylori* show a sharp decrease in AML resistance after freezing related to the down-regulation of genes involved in membrane structure and transport function^29^. Our gastric mucosal specimens are preserved in the brain–heart infusion broth and transported for isolation and susceptibility testing at 4 °C, which may exclude the underestimation of resistance caused by cryopreservation.

There was no significant difference of resistance rates between different gender and age groups in our previous study^10^, consistently with the results in different areas of China^17,28^, but differently with the same areas^28^. Double resistance to CLA and MTZ increased significantly in the 7–12 years and 13–18 years groups compared to 1–6 years group in our current study, whether or not data from 2012–2014 were involved (Table 3, Fig. 2). Then we checked the double resistance to CLA + MTZ in different age groups from 2012 to 2014 and found that it actually increased with age but was not statistically significant, with 8.8% (9/102) in the 1–6 years group, 12.0% (35/291) in the 7–12 years group and 13.2% (20/152) in the 13–18 years group. The number of patients enrolled in Li’s study was also relatively small, which might be the reason of not being able to detect the difference in resistance among different age groups.

The secondary resistance rates were higher than primary resistance in the same population in different regions, especially the resistance rate to CLA^17,19,30,31^. In our study, the primary resistance rate to CLA among the 1513 children without prior *H. pylori* treatment was 31.9%, while the secondary resistance rate among the 125 children with prior treatment was significantly higher (44.8%, *P* < 0.01, Table 3). It was also higher than that in most WHO regions^19^, but lower than that in different regions reported recent years, including Southeast China^17,30,31^.

Multidrug resistance was also an important problem. 37.7% of strains were resistant to more than one antibiotic from 2015 to 2020, 28.7% for double resistance, and 9.0% for triple resistance, which was almost twice to three times compared with that from 2012 to 2014 (19.6%, 16.7% and 2.9% respectively)^10^. The primary resistance rate to CLA, MTZ and LEV was 8.8%, and the secondary resistance rate increased significantly to 15.2% (*P* < 0.05, Table 3). It was higher than that in Southeast China though there were some other patterns of triple resistance including rifampicin^17^. Double and triple resistances were the very important reasons of growing resistance rates (Fig. 1).

With the growing prevalence of global antibiotic resistance in *H. pylori* infection, antibiotic susceptibility testing is becoming increasingly needed for guiding decisions about appropriate therapies in individuals and treatment policies in populations. However, culture-based antibiotic susceptibility testing for *H. pylori*, depending on upper gastrointestinal endoscopy, is not universally available to children in China. On the one hand, pediatric endoscopists and specialized hospitals where gastrointestinal endoscopy is performed not at all hospitals in China. On the other hand, *H. pylori* culture is challenging and culture-based antimicrobial susceptibility testing is not routinely performed in the majority of hospitals. *H. pylori* resistance rates are not systemically monitored by relevant authority institutions, and not yet considered by the department of medicine administration in our hospital. Nowadays, advances in the understanding of basic molecular aspects of drug resistance in *H. pylori* and the development of molecular techniques (such as PCR, next-generation sequencing) have enabled several molecular-based methods for rapid detection of resistance during clinical infections^32^. We had compared partial results of culture-based *H. pylori* diagnosis and antimicrobial susceptibility testing with molecular-based methods (sequencing and gene chip technology) during 2015 to 2016^33,34^. Our results substantiate the sensitivity and accuracy of molecular-based methods to be higher than the culture-based though there are challenges remaining. Importantly, most molecular-based assays can either be culture-based when performed on cultured isolates or culture-free when directly applied on various types of biological specimens such as fresh, frozen or paraffin-embedded gastric biopsy samples, stool samples and gastric juice. Future studies should aim at the latter and are needed for standardization and implementation of easy-to-use computational tools for detecting resistance-related genetic determinants. These should be more efficient to adopt eradication therapies by antimicrobial susceptibility testing in the first line setting in future.

## Conclusions

In current study, we demonstrated very high antibiotic resistance rates of *H. pylori* to CLA, MTZ and LEV in a large number of clinical strains isolated from children from 2015 to 2020 and found linear increases of general antibiotics resistance from 2012 to 2020. Before antimicrobial susceptibility testing for *H. pylori* is available universally, our study might provide reference data for *H. pylori* resistance rates in children in southeast China. Certainly, *H. pylori* resistance to commonly used antibiotics in this region of China is very serious and it is imperative to perform individual treatment based on susceptibility test and choose the relative effective regimens in *H. pylori* eradication therapy.

## Data Availability

All data generated or analyzed during this study are included in this published article.

## Abbreviations

CLA: Clarithromycin
AML: Amoxicillin
MTZ: Metronidazole
FZD: Furazolidone
TET: Tetracycline
LEV: Levofloxacin
CFU: Colony forming unit
CLSI: Clinical and Laboratory Standards Institute
ATCC: American type culture collection
ESPGHAN/NASPGHAN: The European Society for Pediatric Gastroenterology Hepatology and Nutrition/The North American Society for Pediatric Gastroenterology, Hepatology and Nutrition
PCR: Polymerase chain reaction
